# Surface functionalization of microscaffolds produced by high-resolution 3D printing: A new layer of freedom

**DOI:** 10.1016/j.mtbio.2025.101452

**Published:** 2025-01-03

**Authors:** Oliver Kopinski-Grünwald, Stephan Schandl, Jegor Gusev, Ourania Evangelia Chamalaki, Aleksandr Ovsianikov

**Affiliations:** Research Group 3D Printing and Biofabrication, Institute of Materials Science and Technology, TU Wien (Technische Universität Wien), Getreidemarkt 9/308, 1060, Vienna, Austria

**Keywords:** Microscaffolds, Scaffolded spheroids, VEGF, Surface modification, High-resolution 3D printing, Growth factors, Tissue engineering

## Abstract

Scaffolded-spheroids represent novel building blocks for bottom-up tissue assembly, allowing to produce constructs with high initial cell density. Previously, we demonstrated the successful differentiation of such building blocks, produced from immortalized human adipose-derived stem cells, towards different phenotypes, and the possibility of creating macro-sized tissue-like constructs *in vitro*. The culture of cells *in vitro* depends on the supply of various nutrients and biomolecules, such as growth factors, usually supplemented in the culture medium. Another means for growth factor delivery (*in vitro* and *in vivo*) is the release from the scaffold to alter the biological response of surrounding cells (e.g. by release of VEGF).^1^ As a proof of concept for this approach, we sought to biofunctionalize the surface of the microscaffolds with heparin as a "universal linker" that would allow binding a variety of growth factors/biomolecules. An aminolysis step in an organic solvent made it possible to generate a hydrophilic and charged surface. The backbone of the amine, as well as reaction conditions, led to an adjustable surface modification. The amount of heparin on the surface was increased with an ethylene glycol-based diamine backbone and varied between 8 and 40 ng per microscaffold. Choosing a suitable linker allows easy adjustment of the loading of VEGF and other heparin-binding proteins. Initial results indicated that up to 5 ng VEGF could be loaded per microscaffold, generating a steady VEGF release for 16 days. We report an easy-to-perform, scalable surface modification approach of polyester-based resin that leads to adjustable surface concentrations of heparin. The successful surface aminolysis opens the route to various modifications and broadens the spectrum of biomolecules which can be delivered.

## Graphical abstract

**Figure:**
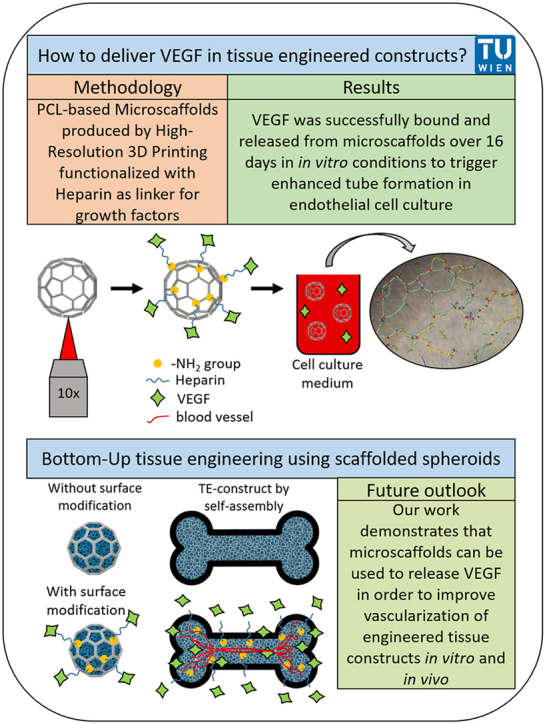

## 1 Introduction

In tissue engineering (TE), two main approaches can be distinguished: scaffold-based and scaffold-free [1]. Scaffold-free strategies rely on building blocks prepared in two-dimensional (2D) or three-dimensional (3D) cell cultures [2]. Common practice in tissue engineering is using 3D cell aggregates, also referred to as spheroids or pellets, as they have the advantage of mimicking the high cell density in native tissue as well as cell-to-cell interaction closer than 2D cell cultures [2,3]. Spheroids also have the advantage of modularity due to their intrinsic properties, such as self-assembly or fusion ability, provided by cell migration. As a result of cell proliferation and migration, multiple spheroids can fuse to produce larger aggregates. Moldovan et al. have shown that assembly and fusion of multiple (differentiated) spheroids can be used to produce larger tissue constructs [4]. Spheroids can be used for various 3D bioprinting applications such as e.g. spheroid skewering on arrays of metallic needles, in a geometrically defined way to fuse into tissue constructs (called kenzan method) [5], or deposited in a 3D manner in supporting self-healing hydrogels to give rise to spheroid fusion for tissue model development [6]. A drawback of using spheroids as building blocks for bottom-up tissue engineering is their poor shape fidelity, making the assembly into a construct of defined volume and geometry troublesome [7].

Scaffold-based TE often relies on different additive manufacturing technologies, also referred to as 3D printing, to produce cell-free scaffolds. 3D printing can be done *via* basic techniques such as FDM [8] and DLP [9], or high-resolution 3D printing techniques like two-photon polymerization (2PP) [7,10,11], depending on the size and scale of the construct. The scaffolds provide 3D structure, mechanical support and protection for seeded cells. With a wide variety of synthetic polymers, degradation, swelling and mechanical properties, such as Young's modulus, can be altered and adjusted depending on the desired tissue. Synthetic polymers often lack cell-polymer interaction and need additional surface treatments to increase this interaction. This can be achieved by surface coating with cell-responsive molecules, like polydopamine [12], or plasma treatment [13,14]. The use of decellularized ECM, albeit modified or not, may help to overcome cell adhesion and cytocompatibility issues. Furthermore, the ECM may contain important components, like growth factors (GF) and other signaling molecules. However, using decellularized ECM in TE applications often comes at the expense of time-consuming decellularization steps and limitations in matching tissue-specific mechanical properties [15,16].

Vascularization is one of the most important sought-after properties in TE applications, especially for large tissue constructs. Limited transport of oxygen, nutrients and metabolites can result in unfavourable conditions (such as hypoxia) leading to necrotic region formation, typically in the core of the tissue construct. Vascularization or pre-vascularization is perceived as a method to overcome these issues, as it can help to improve nutrient and oxygen transport into the implanted tissue construct *in vivo,* leading to an improved tissue integration [17], and has successfully been demonstrated previously [18,19]. Cell-derived material may already contain (vascular) endothelial growth factor (V/EGF), which triggers vessel formation of endothelial cells, such as human umbilical vein endothelial cells (HUVEC). Cell-stimulation *in vitro* is most commonly performed by supplementing the medium with VEGF and other GFs. However, GFs can also be deliverd with the scaffold, e.g. by loading the scaffolds with GFs prior to cell seeding in order to obtain sustained release over the culture time. It was reported that GFs can be bound onto the scaffold *via* bio(active) molecules, like heparin [20,21]. Binding heparin may be advantageous for being able to bind various GF and its potential to deliver a cocktail of GF at different ratios [22,23].

Our research group recently demonstrated a synergetic approach, the third strategy in TE [[7], [24]]. To combine the benefits of scaffold-based, i.e. controllable mechanical properties, and the scaffold-free approaches, i.e. increased cell density and tissue-specific ECM deposition, we introduced microscaffolds (MS) for TE applications. Spheroids were formed inside highly porous MSs, that were produced in a high throughput fashion using 2PP. The MS retained the biological activity of the cells, in terms of spheroid formation, fusion and cell differentiation. The resulting building blocks, referred to as scaffolded spheroids, showed improved shape fidelity during fusion due to decreased compaction. One further advantage arising from the third strategy in TE, is the possibility to combine the release of biomolecules from the scaffold, while exploiting the self-assembly ability of spheroids to engineer larger tissue constructs in a bottom-up approach. While, the concept of bioassembly using scaffolded spheroids has been shown previously [24], here we accomplish the next step involving the binding of a GF (specifically VEGF) onto the MS surface aimed at improving the vascularization of TE constructs, through sustained GFs release.

This study presents our findings about the surface modification of MS produced by 2PP using heparin as a linker to bind and release biomolecules from their surface. The binding and release of VEGF is demonstrated, and the biological activity of surface-bound VEGF is investigated *in vitro*.

## 2 Materials and methods

### 2.1 Materials

1,6-Hexanediamine (HDA), 4,7,10-trioxatridecane-1,13-diamine (TTDA)s, heparin sodium salt, tetrahydrofuran (THF), 2-(N-morpholino)ethanesulfonic acid, and 1,5-diazabicyclo[4.3.0]non-5-en were obtained from Merck. 2-((Benzoyloxy)imino)-1-(4-(phenylthio)phenyl)octan-1-one (OXE-01) and α,ω-PEG-diamine (M_n_ 2000 Da) were obtained from abcr (Karlruhe, Germany). Human umbilical vein endothelial cells (HUVECs, PELOBiotech GmbH) were infected with red-fluorescent protein (RFP)-expressing lentiviral particles at passage 1. Subsequently, RFP-expressing HUVECs were selected (Zeocin resistant) and expanded in endothelial growth medium- 2 (EGM-2) (Lonza, Basel, Switzerland) for later use. In this experiment, HUVECs from passages 5–6 were used.

### 2.2 Methods

#### 2.2.1 Fabrication of microscaffolds

The MS were produced by 2PP as described previously [7,24]. Briefly, photosensitive PCL-based resin (DEGRAD INX) was obtained from BIO INX (Ghent, Belgium), and 3 wt% OXE-01 were added and dissolved in THF at 40 °C. After homogenization, THF was removed at 60 °C and under reduced pressure. The photosensitive resin was transferred to the sample holders, and the photo-crosslinking was performed using a femtosecond-pulsed laser (Light Conversion, Vilnius, Lithuania) operated at 515 nm, a repetition rate of 75.13 MHz and a pulse duration of 122 fs after the microscope objective (UPlanSApo, 10x/0.4 NA, Olympus, Japan). As reported previously, the following parameters were used to produce 15,000 MS in 2 h by aligning 2 × 7 MS per field of view: 400 mW, layer spacing: 1.9 μm, line spacing: 1.3 μm. A custom-written program was used to process the 3D files and control the hardware [25]. The produced MS have fullerene-like geometry, with a diameter of 300 μm and a strut size of 35 μm, and were released from unreacted resin by dissolving it in THF. They resulting MS suspension was stored in THF at RT until further use.

#### 2.2.2 Aminolysis

A 100 ml round-bottom flask was charged with 10,000 MS in 50 ml THF, 50 ml of TTDA:DBN (2:1 mol:mol, 430 mM TTDA) solution in THF were added, and the reaction was stirred for 45 min at RT. The MS were allowed to settle, the supernatant was exchanged with 100 ml fresh TTDA:DBN solution in THF and the reaction continued for 45 min at RT. The MS were allowed to settle and the supernatant removed before washing three times with 100 ml THF and three times with 100 ml 10 mM MES buffer (pH 5.0). The MS were stored in 10 mM MES buffer (pH 5.0) at 8 °C until further use.

#### 2.2.3 Heparin conjugation

A 0.2 mg ml^−1^ heparin solution in 10 mM MES buffer (pH 5.0) was mixed with a NHS and EDC-HCl (100 and 10 mM, respectively) and stirred for 5 min. The resulting solution was added to the MS in a round-bottom flask to reach a MS concentration of 100 ml^−1^ and the reaction was stirred for 18 h at RT. The MS were allowed to settle, decanted, washed thoroughly with 10 mM MES buffer and stored at 8 °C until further use.

Immobilized heparin was quantified using the BlyscanBlue sGAG assay kit (Biocolor Ltd. United Kingdom) according to the manufacturer's SOP. The MS were centrifuged, the supernatant was removed and 1 ml of dye reagent of the kit added. The Eppendorf tubes were put into a 50 ml centrifuge tube and left on a horizontal rolling bank for 1 h. The MS were centrifuged and the supernatant carefully removed. 0.5 ml of dissociation reagent of the kit were added to the MS and vortexed to dissolve the precipitated dye. The obtained solution was transferred into a 96-well plate and the absorbance was measured at 656 nm.

#### 2.2.4 Surface characterization

Attenuated total reflection infrared spectroscopy (ATR-IR) spectra were recorded on a PerkinElmer Spectrum 65 FT-IR spectrometer equipped with a Specac MKII Golden Gate Single Reflection ATR System in the range between 500 and 4000 cm^−1^ with 16 scans per measurement. The samples were fixated with the corresponding tool of the device.

Water contact angle measurements on 2PP-produced sheets were performed using a Krüss DSA30S device using the AVANCE software. Water contact angle of a 2 μl droplet was measured on the raw material, the material after aminolysis and after heparin immobilization.

#### 2.2.5 Mechanical characterization

The mechanical tests were performed using a Microtester device (CellScale, Canada) in compression mode in PBS at RT. Single MS (with or without the surface-bound heparin) were placed under a 1 × 1 mm^2^ platen attached to a 0.30 mm diameter beam and compressed to 30 % of their original size at a compression rate of 1.5 % s^−1^ (5 μm s^−1^). The MS were modelled as full-body spheres to approximate the Young's Modulus of the MS using the Hertz formula until 10 % deformation.

$$F=\frac{4}{3}R^{\frac{1}{2}}∙\frac{E}{1-\upsilon^{2}}∙d^{\frac{3}{2}}$$

With F, R, E, *ν* and d being the recorded force (μN), the radius of the MS, the apparent Young's modulus (Pa), the Poisson ratio and the displacement (μm), respectively. The Poisson coefficient was assumed to be 0.5.

#### 2.2.6 VEGF binding and release Profile

10 heparin-functionalized MS were transferred into a 1.5 ml Eppendorf tube and incubated in 0.5 ml 1 μg ml^−1^ VEGF in PBS (1 % BSA) for 24 h at RT. The MS were allowed to settle, the supernatant was carefully removed, the MS were washed with PBS (1 % BSA) twice, and the release started in 1 ml PBS (1 % BSA). Samples (0.9 ml) of the supernatant were drawn at predetermined time points over 16 days and refilled with fresh solution. Time points (Days 1, 3, 6, 8, 10, 13 and 16) were chosen to simulate cell culture conditions with medium exchange every 2–3 days. VEGF was quantified by ELISA according to the manufacturers SOP. In the following, "VEGF+" refers to MS incubated in VEGF-solution, while "VEGF-" refers to heparin-coated MS.

#### 2.2.7 Serum starvation and priming for tube formation assay

After expansion in EGM-2, cells were serum-starved for 24 h in starvation medium consisting of Dulbecco's modified Eagle's medium (DMEM) including 1 % Penicillin/Streptomycin (Sigma Aldrich, Missouri, USA) and 1 % fetal bovine serum (Life Technologies, Carlsbad, USA) (FBS), followed by 24 h in the same medium in the presence of 20 MS ml^−1^ (VEGF +/−) for the two tested groups.

#### 2.2.8 Metabolic activity

In parallel to the tube formation assay, HUVECs used for the experiment were further kept in culture (2D) in 6 well plates in presence of 20 MS ml^−1^ (VEGF+/−), and their metabolic activity was verified starting on the day of seeding until the end of the tube formation assay using the Prestoblue assay (Invitrogen, Massachusettes, USA). As controls, HUVECs were cultured under the same culture conditions, without any MS, and with non-heparinzed MS (20 MS ml^−1^) using EGM-2 (regular expansion medium). At each time point, the medium was removed (without removing the MS from the wells), and replaced with starvation medium (+10 % Presto blue solution), or EGM-2 (+10 % Presto blue solution) for the controls. The same solution was also incubated in empty wells (for background correction). After incubation for 2 h, the solution was transferred to a flat bottom 96-well plate, and the fluorescence at 590 nm was measured after excitation at 560 nm on a microplate reader (BioTek, Vermont, USA). After normalizing against the empty background signal, the later time points were normalized to the first measured time point after 48 h of starvation (for each group separately VEGF+/−).

#### 2.2.9 Tube formation assay

The bottom of flat-bottom 96 well plates (Greiner, Kremsmünster, Austria) was coated using 50 μL of growth factor reduced Matrigel® Matrix (Corning, New York, USA) per well. The plate was centrifuged at 300 g in a preheated centrifuge to polymerize for 60 min at 40 °C during centrifugation. 3 MS (VEGF+/−) were placed in individual Eppendorf tubes before 10,000 primed HUVECs (VEGF +/−) were added in 100 μL starvation medium and resuspened for thorough mixing. This cell/MS mixture was transferred into the prepared Matrigel-coated wells.

Images of the tube formation were taken 4, 8, 24, 32 and 50 h after seeding on the Matrigel surfaces, using the inverted microscope (Echo Revolve, San Diego, USA), 4× objective (Olympus, Tokyo, Japan) at three randomly selected field of view per well. Exported images were analyzed using the "Angiogenesis Analyzer" [26] an ImageJ macro software. The number of master junctions, total tube length and total mesh area were analyzed.

#### 2.2.10 Data analysis and statistical analysis

Data processing and plotting were performed in Origin (OriginLab Version 2023b) and in Prism (GraphPad, Version 8.0.2).

## 3 Results and discussion

### 3.1 Surface modification

**Scheme 1:**
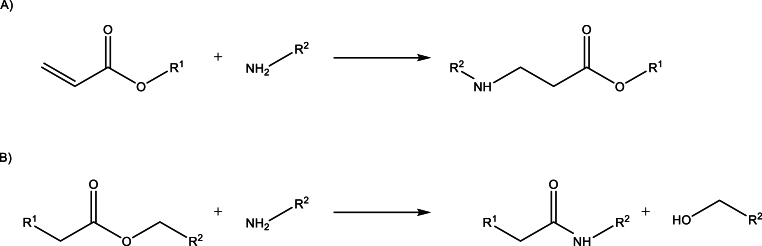
Two reaction pathways are possible to introduce amino groups under the investigated conditions. A) Aza-Michael addition of the primary amine on the α,β-unsaturated carbonyl group provided by unreacted acrylate end groups of the prepolymer. B) Aminolysis of the ester group on the backbone of the prepolymer leading to an alcohol and an amide. The stability of the formed amide hinders the backreaction.

The introduction of amino groups onto the surface of scaffolds composed of either linear or crosslinked polyester can be achieved by aminolysis [[27], [28], [29]] or aza-Michael addiditon [30]. The latter is only an option for unreacted acrylate endgroups, the former is possible for every ester (Scheme 1A) [30,31].

Aminolysis refers to the cleavage of the ester group by simultaneously forming an amide. Due to the stability of the amide group, the backward reaction is not favourable (Scheme 1B). For scaffold modifications, the aminolysis of linear polyester is mostly performed under mild conditions in anti-solvents of the polyester, e.g. 1- and 2-propanol in the case of PCL, and without any catalysts to avoid degradation of the polyester [29,32]. Autocatalyzed aminolysis of PCL in 2-propanol yields low surface concentrations of free amino-groups [32]. Furthermore, the quantification of free surface amino groups can be performed by exploiting the reaction of the amine with ninhydrin, followed by the dissolution of the material and spectroscopic analysis [32]. However, for linear polyacrylates, harsh conditions and strong and toxic reactants, like n-butyl lithium or sodium methanolate, are commonly used [30,31]. When working with crosslinked polyesters, dissolution of the material is not possible. Hence, surface-bound heparin was quantified during the optimization of our protocol. Obtained results suggest that acidic and autocatalytic conditions in 1-propanol did not yield sufficiently high amounts of surface functionalization for crosslinked polyester (see Table 1). Surface-bound heparin of 100 MS was below the limit of detection of the Blyscan Blue sGAG kit.

**Table 1:** Screening of aminolysis reaction conditions analyzed by surface-bound heparin.

	Diamine	Concentration (mM)	Catalyst	concentration (mol%)	Hep (ng)/scaffold
1^31^	HDA	430	None	–	0
2		430	AcOH	20	0
3		430	TEA	0.2	0
4		430	DBN	2	1.6 ± 0.1
5		60	DBN	50	0.8 ± 0.1
6		430	TBD	50	0.1 ± 0.2
7	TTDA	430	DBN	50	19.4 ± 0.8
8	PEG-diamine	6	TBD	50	8.1 ± 0.3

**Fig. 1:**
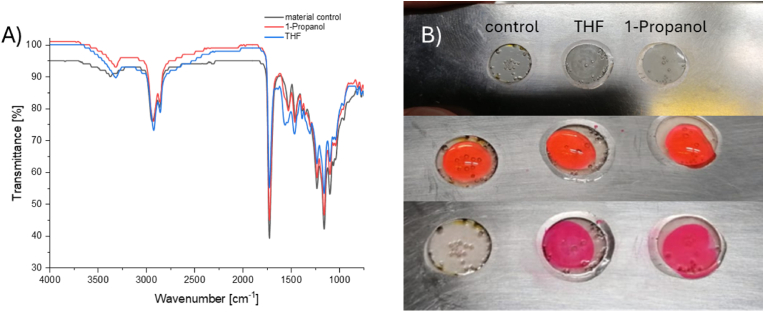
A) FT-ATR-IR spectra from the material control (black) and after the surface modification performed in 1-propanol (red) and THF (blue). The peak at 1567 cm-1 indicates the presence of primary amides formed during the aminolysis reaction. Due to the distinct presence of this peak in THF, this solvent was chosen for the modification of MS; B) Preliminary study on the surface modification on thin films using TTDA and DBN as reactive component in 1-propanol and THF as well as a material control. Before (top), during (middle) staining and after washing (bottom). (For interpretation of the references to colour in this figure legend, the reader is referred to the Web version of this article.)

The low degree of surface modification further caused aggregation of the MS due to non-polar interactions in aqueous medium. After successful surface modification, amino groups should introduce surface charges and increase hydrophilicity. This proved that protocols reported in the literature are ineffective and could not be used for crosslinked polyesters. Changing the reaction conditions from autocatalyzed in 1-propanol to base-catalyzed in THF led to significantly increased surface modification, as seen in the ATR-FTIR spectra of treated disks (Fig. 1A). A peak at 1567 cm^−1^ indicates the presence of primary amides formed during the aminolysis reaction. Staining with acidic dye Eosin Y (Fig. 1B) in aqueous conditions proved the presence of available amines on the surface of the material after the surface modification. Free amines are essential for the following reaction steps to conjugate heparin onto the material.

It was also found that the reaction proceeded with higher yields when the reaction solution was exchanged once during the reaction, increasing the surface-bound heparin content from 15 to 25 ng per MS. Additionally, the backbone of the diamine drastically influenced the yield of the reaction. Comparing HDA and TTDA, it was found that the latter was more efficient (see Table 1), indicating that the diethylene glycol core of TTDA is stabilized in aqueous solution by hydration, enabling higher reactivity with activated heparin. Extending the ethylene glycol backbone to an α,ω-PEG-diamine (M_n_ 2000 Da) did not increase the yield further. It was also observed that the amount of heparin that can be conjugated to the surface was directly proportional to the concentration of TTDA used during the aminolysis reaction (Fig. 3A). Although 800 mM TTDA in THF resulted in the highest heparin loading, the handling of the MS in solution was negatively affected.

**Fig. 2:**
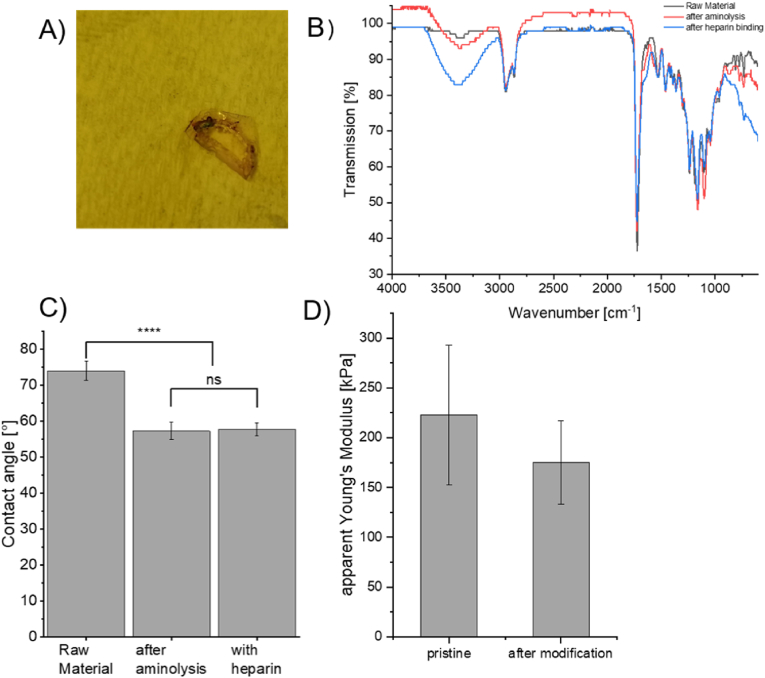
A) Staining of heparin on material discs for preliminary tests. The blue areas indicate the binding of the dye to the heparin, which was bound to the material's surface. B) ATR-IR spectrum of the surface after heparin conjugation. It can be seen that the transmittance at wavenumber above 3000 cm^−1^ is further decreased, indicating more available -OH and -NH_2_ groups introduced during the heparin-binding. The increase in signals in the fingerprint area (below 1000 cm^−1^) further suggests the introduction of heparin, which is distinctly different from the material. C) The change of contact angle of water on a material film. Although already hydrophilic as raw material, introducing amino groups on the surface further increases surface hydrophilicity. The binding of heparin did not lead to a further change in contact angle. D) Apparent Young's modulus was calculated before and after surface modification. (For interpretation of the references to colour in this figure legend, the reader is referred to the Web version of this article.)

Surface-immobilization of heparin was first studied on material disks (Fig. 2A) using 2 mg ml^−1^ heparin and 50 and 5 mM NHS and EDC-HCl, respectively. The ATR-IR (Fig. 2B) further confirmed that heparin was covalently bound to the previously introduced amino groups by a lower transmittance above 3000 cm^−1^, indicated by the presence of more -OH and -NH_2_ groups. The presence of heparin did not further change the water contact angle after the initial drop caused by the introduction of surface-amino groups (Fig. 2C). Mechanical characterization of single MS with and without heparin-modification was performed. The comparison of the calculated apparent Young's moduli of MS with and without heparin-modification did not reveal any statistically significant difference between the two groups (Fig. 2D). Therefore, it was concluded that the surface modification did not affect the mechanical properties of MS and that the detailed mechanical analysis of PCL-based photosensitive resin, are valid [10]. The reduction of the Young's modulus between the bulk material, as tested in UV-cured tensile test specimen can be explained by our assumption outlined in section 2.2.5 in combination with the high porosity (96 %) of the MS' structure [10]. For the clinical applications of MS, the assessment of fracture or impact toughness of the material remains an important prerequisite, which is not presented here and will be included in future work. However, it is worth mentioning that the MS would not be used as sole placeholder but rather in combination with cellular spheroids. In our previous work [7,24] wedemonstrated that spheroids cultured in MS, indeed can produce tissue-specific ECM. This additional ECM is of course associated with altered mechanical properties in *in vitro* or *in vivo* conditions (as the ECM could take up part of the mechanical load). Due to these reasons, this study focuses only on the effective surface modification and does not go into deeper mechanical characterization and analysis, as here the concept of surface functionalization as universal approach useful for different tissue types is presented.

**Fig. 3:**
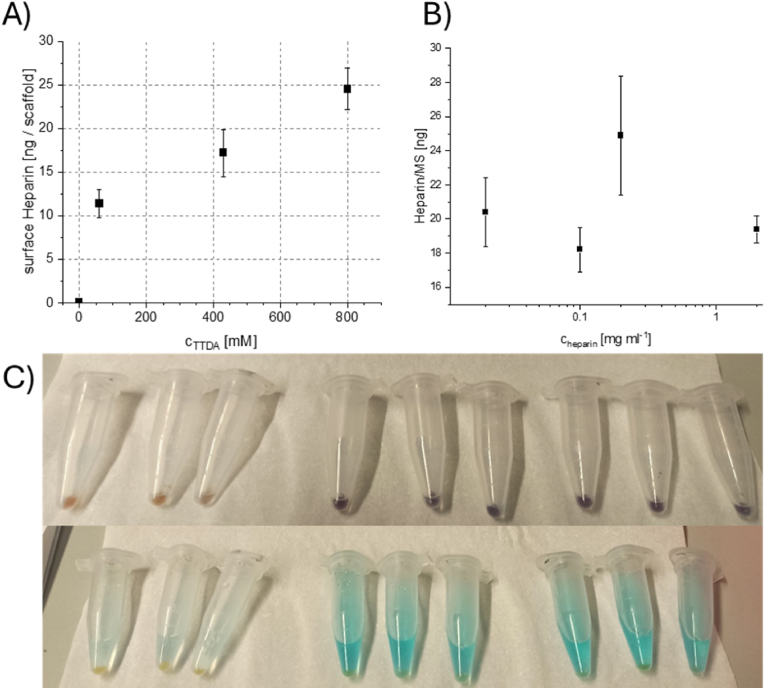
Quantification of heparin per MS as (A) function of TTDA concentration during the aminolysis and (B) function of heparin concentration in the conjugation step. C) 100 MS in a 1.5 Eppendorf tube after staining with 1,1-dimethylene blue (top) and after the release of bound dye (bottom) with increasing surface concentration of heparin (left to right). (For interpretation of the references to colour in this figure legend, the reader is referred to the Web version of this article.)

Screening the heparin concentrations revealed that the necessary concentration to saturate all introduced surface amino groups was below 0.02 mg ml^−1^, as shown in Fig. 3B. Increasing the concentration to 2 mg ml^−1^ for 100 MS ml^−1^ in the reaction did not significantly affect the yield of surface-bound heparin but peaked at 0.2 mg ml^−1^. MS treated at this heparin concentration showed the best handling performance as the lowest amounts of aggregates were obtained, and MS did not stick to the walls of the reaction vessel.

It was found that, under these conditions, the maximum load of heparin reached 30 ng per MS. Heparin was also found on MS without coupling agents; however, it was loosely bound to the MS due to charge-charge interaction. Heparin, a polysaccharide and a polyelectrolyte, can form salts *via* the carboxylates and sulfonates with the primary amines on the scaffolds. The biomolecule can then align on the surface and be released with thorough washing steps. In negative control experiments without EDC-NHS as coupling agents, heparin on the MS decreased over time during sorting (data not shown), indicating the necessity for the coupling agent. Optimizing the aminolysis and heparin-binding protocols led to a highly reproducible, robust and scalable surface modification for polyester-based resins used in 2PP processing for the modifications of MS (as indicated in Fig. 3C). The chemistry of the presented protocol allows the immobilization of various biomolecules *via* EDC-NHS chemistry, or further functionalization atop the present amino groups, e.g. grafting of new polymer chains onto the surface. Furthermore, we report the high-throughput modifications of up to 20,000 MS in one modification step.

### 3.2 VEGF release from microscaffolds

**Fig. 4:**
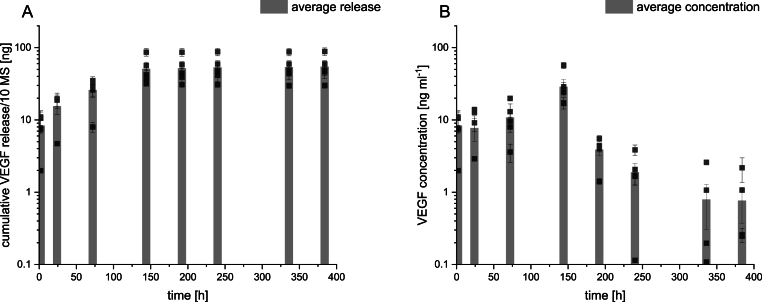
Cumulative VEGF release (A) and measured VEGF concentration per time point (right) of 10 MS. The measured concentration at a given time can be interpreted as the global concentration of VEGF in solution. The concentration of VEGF increased steadily until day 6 before the release was reduced almost but a steady concentration of 1 ng ml^−1^ was obtained until the end of the release study.

As heparin is vital in regulating angiogenesis and other processes closely related to tissue regeneration, the biomolecule is often used for surface modifications [22] or as an additive during scaffold production [33]. Heparin is used as a VEGF delivery system to bind and stabilize VEGF dynamically [34,35]. In this case, the heparin binding domain of VEGF is exploited to bind the growth factor to the biomaterial's side chain reversibly. Contrary to other reported VEGF-delivery systems, in our system, the quantity of VEGF released can be adjusted by the surface concentration of heparin on the MS. The concentration of free VEGF in solution is, in turn regulated by thermodynamics by cleavage of the heparin-growth factor bond, which allows the release of VEGF from the MS. With 10 MS per ml in solution, a peak VEGF concentration and an average concentration over 6 days of 28 and 14 ng ml^−1^, respectively, were obtained. The release of VEGF was sustained for 16 days in culture and a cumulative amount of 55 ± 22 ng were released per 10 MS (see Fig. 4). In order to benchmark our results, Table 2 summarizes previously reported findings of VEGF-releasing heparinized scaffolds. Due to the lack of uniformity of presented results in literature, a direct comparison between each delivery approach was barely possible. Nevertheless, Table 2 attempts to benchmark our heparin-based system and other VEGF delivery reports.

**Table 2:** The heparinized MS-based VEGF delivery system outperforms all heparin-based delivery systems. Weight of an individual MS was calculated by weighing a batch of 10,000 MS before surface modification and divided by 10,000. The surface area was obtained through the CAD file and the volume was calculated from the radius of the MS structure. "Released within" refers to the time of release in which 90% on the cumulative release was found. Results from literature estimated from reported figures. N/A = not available, Hep- = heparinized.

Material	VEGF per scaffold (ng/mg)	Bound VEGF per volume (ng cm^−3^)	Bound VEGF per area (ng cm^−2^)	Released within (d)
Hep-MS	3333	3.9⸱10^5^	1410	5
Hep-PLLA/PCL [21]	N/A	2200	N/A	2
Hep-Collagen [36]	400	N/A	N/A	N/A
Hep-Collagen [23]	1000	N/A	N/A	N/A
Hep-PCL + ECM [20]	1.36	N/A		10
Hep-Collagen [37]	35	1650	N/A	∼20
PCL/chitosan [38]	N/A	N/A	300	∼10
Hydroxyapatite [39]	1.8	N/A	N/A	7

**Fig. 5:**
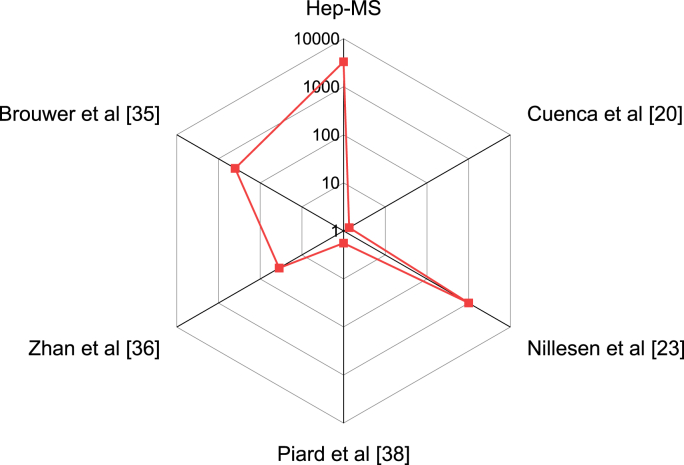
Spider chart depicting the amount of VEGF bound to scaffold in [ng mg^−1^], VEGF biding of microscaffolds outperforms, previous published results of VEGF-releasing heparinzed scaffolds.

Comparison with previously reported heparinized scaffolds, led to the conclusion that the here reported method leads to 3–3000x higher amount of VEGF bound to the scaffolds surface (ng/mg) and ∼177x increased bound VEGF per volume when compared to previous reported results (see Table 2 and Fig. 5). This circumstance can be explained by screening of the aminolysis reaction conditions leading to an efficient heparinization of the MS surface responsible for effective VEGF binding and release. Furthermore, the geometry of the MS itself plays a role for the effective loading and release of GFs, as e.g. 20 MS have a surface to volume ratio of ∼15.4 mm^−1^, while a cuboid bulk material with the same volume would have a volume to surface ratio of only ∼9.09 mm^−1^. A effect that is even more pronounced when increasing the total volume.

After quantifying the VEGF release from MS, we investigated the bioactivity of the released VEGF from MS in a tube formation assay, which can be considered the standard *in vitro* assay to study biomolecule's influence on angiogenesis [[40], [41], [42]]. It is worth mentioning that a global concentration of VEGF in the solution above the MS is measured during the ELISA measurements. Under static conditions, the concentration of VEGF around the MS is determined by diffusion of VEGF. In the first step, VEGF debinds from heparin, and in the second step, it diffuses freely into the solution. This causes a VEGF gradient originating from the proximity of the MS. Therefore, the concentrations of VEGF in the immediate proximity of the MS could reach levels well above what was measured in the ELISA. However, due to the close proximity (less than 150 μm) of the cells within the MS, and its small diameter, this VEGF gradient is most likely negligible during the *in vitro* culture and cells will experience a constant concentration of VEGF. The VEGF gradient outside of the MS (especially in the case of implantation of multiple scaffolded spheroids with high packing density) could attract host vascular endothelial cells towards the implant, to trigger vascularization of the implanted structure.

**Fig. 6:**
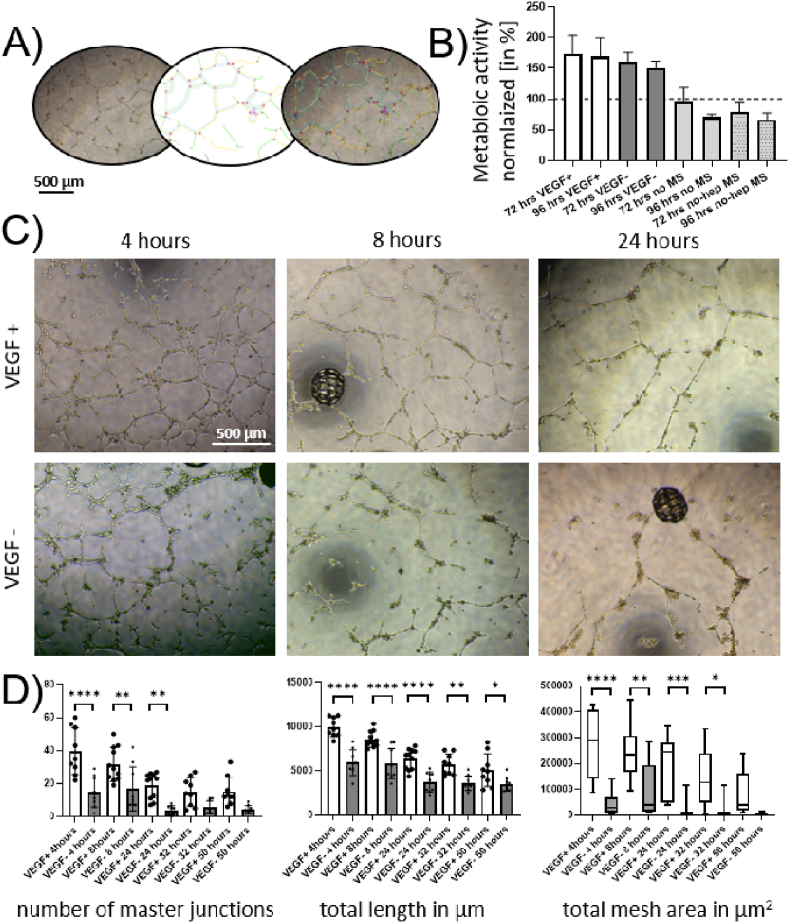
Effects of basal DMEM preconditioned using MS loaded with/without VEGF (VEGF+/−). A) Graphical representation of the image analysis using ImageJ; B) metabolic activity measured during serum-starvation (normalized to the first timepoint = 48 h after starting of serum-starvation/culture = dotted line); C) Representative microscopic images depicting the tube formation progression from 4 to 24 h after seeding on the Matrigel surface; For both tested groups, D) Quantification of the number of master junctions, total tube length, and total mesh area covered by the tubular network.

A standard sprouting assay on Matrigel was designed to study the influence of the VEGF released from the heparin-modified MS. To realize the assay, a starvation medium with 20 MS per ml (VEGF+/−) was used for serum starvation before seeding 10,000 HUVECs per well (in the presence of 3 VEGF+/- MS) onto the Matrigel surfaces. It can be concluded that both tested groups showed successful tube formation due to cellular migration and rearrangement (Fig. 6C). Quantification using image analysis software (schematically explained in Fig. 6A) unveiled that the number of master junctions formed in the presence of VEGF+ MS was significantly higher for up to 24 h, while the analyzed total tubular length remained significantly higher for each tested time point (up to 50 h after cell seeding), when compared to the VEGF- group. Furthermore, the total mesh area covered by the tubular network was significantly higher in the VEGF+ group for up to 32 h after seeding when comparing individual time points between the two tested groups. Therefore, it can be concluded that the VEGF release from MS cultured with HUVECs reached a sufficient level, as indicated by ELISA results, to stimulate the tube formation of HUVECs. Lee Hyunsook and Kang Kyu-Tae reported the influence of increasing VEGF concentrations on human endothelial colony-forming cells in tube formation assay in 2018. From their publication, it can be derived that increasing VEGF concentrations during the tube formation leads to an increase in tubular length after 24 h, while a tube depletion in the absence of VEGF is observed simultaneously [43]. The same trend was observed in our study when comparing images for VEGF+ and VEGF- groups in Fig. 6D (24-h timepoint), as prolonged tube lengths (in the VEGF+ group) were detectable, and a tube depletion in the VEGF- group was observed at the same time scale. Taken together, these findings go well in accordance with previous work and further prove that the VEGF release from MS reached a biologically relevant concentration to improve the tube formation abilities of HUVECs.

The VEGF concentration in the preconditioned medium caused rapid and sustained sprouting, which is statistically significantly increased compared to basal medium up to 50 h of culture time.

### 3.3 Metabolic activity during starvation

Analysis of the metabolic activity revealed that neither after 48 h of starvation nor for the subsequent days, a significant difference between the VEGF+ group compared to the VEGF- group was detectable (see Fig. 4B). Interestingly, the metabolic activity increased with the duration of serum starvation. A possible explanation for this behaviour was published in 2023 by Mario Lorenz et al. [44], who found that serum starvation in endothelial cells can accelerate the intracellular metabolism (also observed *in vivo*) as endothelial cells need to remain metabolic active, especially in unfavourable conditions (e.g. *in vivo*, during traumatic injuries etc.). The accelerated intracellular metabolism could explain the measured increase in metabolic activity during serum-starvation and further indicate a "successful" serum-starvation of the used cells. It has to be noted here that serum-starvation prior tube formation assay is recommended in the literature [44,45], and applied in previous publications studying biomaterial or biomolecule's influence on angiogenesis in tube formation assays [45]. HUVECs cultured under regular expansion conditions (in EGM-2) showed decreasing metabolic activity, most likely as they reached confluency after the first 48 h of the experiment. However, there was no statistically significant difference between the control groups cultured in the presence of non-heparinzed MS and the group cultured without any MS (for each time point). From this direct comparison, we conclude that the MSs are cytocompatible, as no negative effect on the metabolic acitivty could be detected (when compared to controls without any biomaterial). This experiment further confirms results on cytotoxicity published earlier using MSs and human adipose derived stem cells, where decreasing metabolic activity for both material (without surface modification) and control group was observed in 3D^7^.

## 4 Conclusion

We report the successful surface modification of MS produced by 2PP within the third strategy of tissue engineering concept. A two-step procedure was demonstrated, which could be upscaled by modifying up to 20,000 scaffolds simultaneously. Within the first step, base-catalyzed aminolysis of the PCL-backbone led to the introduction of free primary amino groups on the surface of these MS. Through these, the MS were freely dispersible in aqueous solutions, leading to the evasion of organic solvents for handling the MS. In the second step, heparin was successfully conjugated to the scaffold surface *via* EDC-NHS chemistry. Through the heparin, we could bind and release VEGF in cell culture, leaving the scaffolds' mechanical properties unaltered. Concentrations of released VEGF reached 10–30 ng ml^−1^ after three days of incubation, causing a strong response of HUVECs cultured on Matrigel. Although the VEGF concentration in the immediate microenvironment was not accessible, we report a proof of concept for scaffold-based growth factor delivery through biofunctionalization of MSs produced by 2PP. Besides VEGF, other growth factors, such as TGF-β or FGF, could be immobilized, using the described surface modification method, as both growth factors are known to have heparin-binding properties such as the here demonstrated VEGF. Single GF or multiple GFs could be immobilized and released during culture to prime cells towards certain phenotypes (tissue-specific), in accordance with the already established method of scaffolded spheroids that are differentiated *in vitro*. While heparin was the initial target for the surface modification, the successful introduction of surface-bound amino groups offers many opportunities. Due to the mild reaction conditions and availability of various NHS-ester derivates of biomolecules, it is possible to immobilize those biomolecules. Furthermore, these amino groups can be used to directly graft new polymers, e.g. *via* ring-opening polymerization, or introduce new polymerizable groups, such as acrylates or epoxides. These options show the great potential of the presented surface modification for the presented VEGF delivery and other TE applications. Furthermore, the conjugation of biotin could further increase the portfolio of biomolecules that bind to the surface. In conjunction with the architectural freedom given by high-resolution printing, the protocol could elevate fundamental research in 3D cell culture.

## CRediT authorship contribution statement

**Oliver Kopinski-Grünwald:** Writing – original draft, Visualization, Methodology, Investigation, Formal analysis. **Stephan Schandl:** Writing – original draft, Visualization, Methodology, Investigation, Formal analysis. **Jegor Gusev:** Validation, Investigation. **Ourania Evangelia Chamalaki:** Investigation, Validation. **Aleksandr Ovsianikov:** Writing – review & editing, Supervision, Resources, Funding acquisition.

## Declaration of competing interest

The authors declare the following financial interests/personal relationships which may be considered as potential competing interests:Aleksandr Ovsianikov is co-founder of UpNano GmbH, a TU Wien spin-off active in the area of two-photon polymerization. His current relationship with UpNano includes: consulting, advisory and equity. The rest of the authors declare that they have no known competing financial interests or personal relationships that could have appeared to influence the work reported in this manuscript.

## Data Availability

Data will be made available on request.
